# Metallic glassy Zr_70_Ni_20_Pd_10_ powders for improving the hydrogenation/dehydrogenation behavior of MgH_2_

**DOI:** 10.1038/srep26936

**Published:** 2016-05-25

**Authors:** M. Sherif El-Eskandarany

**Affiliations:** 1 Nanotechnology and Advanced Materials Program, Energy and Building Research Center, Kuwait Institute for Scientific Research Safat 13109, Kuwait - State of Kuwait

## Abstract

Because of its low density, storage of hydrogen in the gaseous and liquids states possess technical and economic challenges. One practical solution for utilizing hydrogen in vehicles with proton-exchange fuel cells membranes is storing hydrogen in metal hydrides. Magnesium hydride (MgH_2_) remains the best hydrogen storage material due to its high hydrogen capacity and low cost of production. Due to its high activation energy and poor hydrogen sorption/desorption kinetics at moderate temperatures, the pure form of MgH_2_ is usually mechanically treated by high-energy ball mills and catalyzed with different types of catalysts. These steps are necessary for destabilizing MgH_2_ to enhance its kinetics behaviors. In the present work, we used a small mole fractions (5 wt.%) of metallic glassy of Zr_70_Ni_20_Pd_10_ powders as a new enhancement agent to improve its hydrogenation/dehydrogenation behaviors of MgH_2_. This short-range ordered material led to lower the decomposition temperature of MgH_2_ and its activation energy by about 121 °C and 51 kJ/mol, respectively. Complete hydrogenation/dehydrogenation processes were successfully achieved to charge/discharge about 6 wt.%H_2_ at 100 °C/200 °C within 1.18 min/3.8 min, respectively. In addition, this new nanocomposite system shows high performance of achieving continuous 100 hydrogen charging/discharging cycles without degradation.

Hydrogen is an energy carrier holds tremendous promise as a new clean energy option in future energy systems^1^. Hydrogen storage, which cuts across both hydrogen production and hydrogen applications and thus assumes a critical role in initiating a hydrogen economy, has been the subject of intensive research for many years. However, hydrogen can be stored as compressed gas cylinders under very high pressure (~350 bar) or as liquid at −253 °C, employing these storage approaches in real applications are difficult due to the high cost and safety issues^1^. Apart from these traditional ways of hydrogen storing, Mg and Mg-based materials have been considering as the most candidate hydrogen storage materials for real applications^2^,^3^,^4^,^5^,^6^,^7^,^8^. The worldwide interest on Mg metal is attributed to its natural abundance, light weight, and its capability to store hydrogen up to 7.60 wt.% (0.11 kg H_2_L^−1^). In spite of these attractive properties of MgH_2_, and the simple way used for producing it in an industrial scale via reactive ball milling (RBM) technique^9^,^10^, MgH_2_ in its pure form has a high stability and shows very slow kinetics of hydrogenation dehydrogenation at temperatures less than 400 °C. Such serious drawbacks are considered as major barriers preventing such an attractive metal hydride material for potential use in fuel cell applications^2^,^7^,^11^,^12^. Within the last two decades, enormous efforts have been dedicated in order to improve the hydrogenation/dehydrogenation behaviors of MgH_2_ (see for example refs 7,13,14) throughout two three strategies.

The philosophy of the first strategy depends on introducing a heavy network of lattice imperfections and defects to Mg, pure MgH_2_, and Mg-based alloys without using any types of catalysts^15^. This mechanical treatment regime has been successfully achieved by subjecting the powders to a long-term of a high-energy ball milling runs^16^ (can be extended to several hundreds of hours) through a mechanically-induced cyclic phase transformations^17^. We should emphases that β-γ and cyclic β-γ-β^18^ phase transformations are always coupled with drastic decreasing in the grain sizes of MgH_2_ powders. Since the hydrogen diffusion along grain boundaries is much faster than diffusion in side grains^19^, the hydrogenation/dehydrogenation kinetics of MgH_2_ are outstandingly improved upon producing such fine nanostructured grains, containing a large number of grain boundaries (see for example refs 16 and 18). Severe plastic deformation, using cold rolling^20^, equal channel angular pressing, and high-pressure torsion multiple forging or cyclic, channel die compression techniques (a summary of these techniques are summarized in ref. 21) are the other options used for refining the coarse MgH_2_ grains to obtain micro-scaled structured grains. It has been pointed out by Dufour and Huot^22^ that as-cold rolled Mg–Pd alloy shows fast kinetics when compared with as-ball milled samples. They attributed this improvement seen in the bulk cold- rolled samples to its resistance to the air contamination^22^.

The second strategy used for enhancing the kinetics behavior of MgH_2_ depends on doping the metal hydride powders with selected catalysts/nanocatalysts to form ultrafine nanocomposite powders with advanced hydrogenation/dehydrogenation properties. Huge number of nanocatlysts such as pure metals^23^, intermetallic compounds^24^,^25^, metal oxides^26^, metal/metal oxide nanocomposite^27^, metal crbides^28^, metal chlorides^29^. Recently, two review articles have been published discussing the role of doping MgH_2_ particles with catalytic agents on improving the kinetic behavior and cycle-life time^30^,^31^.

Almost all of these reported systems when compared with pure MgH_2_ nanocrystalline powders, they significant usage merits, indexed by lower hydrogen sorption/desorption temperatures and faster hydrogenation/dehydrogenation kinetics,. Moreover, some of these nanocomposite systems such as MgH_2_/7 Mn_3.6_Ti_2.4_^25^, MgH_2_/5Ni/5Nb_2_O_5_^27^, MgH_2_/5TiC^28^, and MgH_2_/10 big-cube Zr_2_Ni^32^ powders have shown high performance cyclability for achieving complete 1000, 600, 696 and 2546 complete cycles at hydrogenation/dehydrogenation temperature in the range between 250–275 °C without serious hydrogen storage degradations. In addition, these interesting systems have shown fast absorption/desorption kinetics at relatively low temperature (250–275 °C) ranging between 41 to 120 s/121 to 613 s, respectively with reasonable hydrogen storage capacity ranging between 5.1 to 5.3 wt.%. It is believed that high energy ball milling MgH_2_ with the nanocatalyst powder of carbides, oxides, and intermetallic abrasive powders lead to fast grain refining of the MgH_2_ upon releasing the crystalline stored energy, leading to refine the MgH_2_ grains along their grain boundaries and to produce fine grains. Such fine grains with their short-distance grain boundaries always facilitate short diffusion path, leading to fast diffusion of the hydrogen atoms^33^,^34^.

A very attractive and unique approach used to improve the kinetics behaviors of MgH_2_ without using expensive metallic catalysts has been proposed by Jeon *et al*. in 2011^35^. In their novel process, Mg nanocrystals (NCs) were embedded into polymer (PMMA) matrix in an inert gas atmosphere to form air-stable Mg NCs/PMMA nanocomposite material. Encapsulation the nanosized Mg (~5 nm in diameter) in a polymer with selective gas permeability, protecting the NCs from O_2_ and H_2_O. This contamination-free system enjoys high density of hydrogen (6 wt% of Mg) and rapid kinetics (loading in <30 min at 200 °C).

In the last strategy employed for enhancing the hydrogenation/dehydrogenation properties of MgH_2_ is to melt pure bulk Mg with selected alloying elements such as Ni, Pd, and Nd to obtain less stable binary, ternary or multicomponent Mg-based alloy systems with lower heat of formation (ΔH^for^). In many cases, the synthesized alloying Mg-based alloy systems do not show attractive properties when compared with MgH_2_. For example, alloying Mg with Ni to form binary Mg_2_Ni lowers the ΔH^for^ of the metal hydride phase (Mg_2_NiH_6_) to −64.5 kJmol^−1^ instead of −74.5 kJmol^−1^ for pure MgH_2_. However, the system shows a dramatic degredation in its hydrogen storage capacity (3.5 wt.%) with no significant decreasing in the decomposition temperature^36^. In 2001, Yamada *et al*.^37^ proposed Mg-rich (90 at.%) systems of Mg-Pd, Mg-Nd and Mg-Pd-Nd to replace the traditional Mg_2_Ni alloy. They reported that Mg-based system containing Pd show at 300 °C PCTs curves with three plateau-like regions and hydrogen absorbency of 5 wt.%. They pointed out that the hydrogenation/dehydrogenation of Mg-Nd system was influenced by a catalytic effect of the formed NdH_2.5_ and NdH_3_ phases that assisted hydriding and dehydriding of the Mg matrix. For Mg-Pd system, they investigated that disproportional reaction of Mg_6_Pd to form Mg_5_Pd_2_ and MgH_2_ retarded the overall reaction kinetics. In spite of their efforts dedicated to introduce a new Mg-based alloy system with advanced kinetics behavior, their proposed Mg_89_Pd_7_Nd_4_ alloy required 50 min to absorb about 4 wt.% H_2_ at 300 °C. In addition, the dehydrogenation process of this system required 150 min at the same operating temperature to be completely achieved^37^.

In 2009, Ouyang *et al*.^38^ demonstrated a complete study of Mg_3_Pr and Mg_3_PrNi_0.1_ hydride systems. Based on their results, both alloys showed reversible hydrogenation (290 °C/35 bar H_2_)/dehydrogenation (290 °C/0.003 bar) cycles. Under these conditions, Mg_3_Pr and Mg_3_PrNi_0.1_ exhibited storage capacities of 2.58 and 3.23 wt.%, respectively. More recently, *in situ* formation of cycle stable CeH_2.73_-MgH_2_-Ni nanocomposites, from the hydrogenation of as-melt Mg_80_Ce_18_Ni_2_ alloy, with excellent hydrogen storage performance has been proposed in 2014 by Ouyang *et al*.^39^. This nanocomposite system demonstrated reversible hydrogen storage capacity of 4.0 wt %, at a low desorption temperature (232 °C) with fast kinetics and long cycle life (500 cycles). Moreover, CeH_2.73_-MgH_2_-Ni nanocomposite exhibited a low apparent activation energy of 63 kJ/mol.

In 2011, In was used as an alloying element to form Mg_0.95_In_0.05_ solid solution, using mechanical alloying (MA) technique^40^. Fully reversible transformation in the Mg–In–H system with reduced hydrogen sorption reaction enthalpy was demonstrated. The hydrogen storage capacity of Mg_0.95_In_0.05_ solid solution measured at 30bar/300 °C showed a high value of 5.5 wt.%^40^. In a different proposed by the same school^41^, elemental In was replaced by Sn with the same atomic percent (0.05) to form Mg_0.95_Sn_0.05_ solid solution, using MA technique. They reported that Mg–5at.% Sn nanocomposite exhibited elevated plateau pressure and destabilized thermodynamics due to the introduction of large amount of interface energy in Mg/Mg_2_Sn nanocomposite. However, the hydrogen storage capacity measured at 30 bar/323 °C was lower (4.8 wt.%) than Mg-In system. The corresponding hydrogenation (50 bar/362 °C)/dehydrogenation (0.003 bar/300 °C) kinetics, for Mg–5at.% Sn system were 170 and 130 min, respectively^41^.

Further efforts dedicated for lowering the stability of MgH_2_ system was achieved by Cao and his team by introducing elemental Al and Ti into Mg(In) solid solution to form Mg_85_In_5_Al_5_Ti_5_ alloy, using plasma milling technique^42^. They claimed that addition of Ti and *in-situ* synthesized MgF_2_ improved the kinetics and the introduction of In as well as Al imparted enhanced thermodynamics to the Mg_85_In_5_Al_5_Ti_5_ system^42^. In their study, the dehydrogenation enthalpy change and activation energy of this new system were 65.2 kJ/(mol H_2_) and 125.2 kJ/mol, respectively. The hydrogen storage capacity this system measured at 30 bar/340 °C was 5.5 wt.%.

Apart from such a long list of traditional catalysts used for improving the MgH_2_, here we show, for the first time the effect of employing a small mole fractions (5 wt.%) of Zr_70_Ni_20_Pd_10_ metallic glassy powders on destabilizing the MgH_2_ and improving its kinetics. Theas-synthesized nanocomposite MgH_2_/5 wt.% Zr_70_Ni_20_Pd_10_ powders, which shows high density of hydrogen, possess advanced hydrogenation/dehydrogenation processes taking place at low temperature and very short of time.

## Results

### Structure and morphology

#### Amorphous Zr_70_Ni_30_Pd_10_ powders

**Figure 1:**
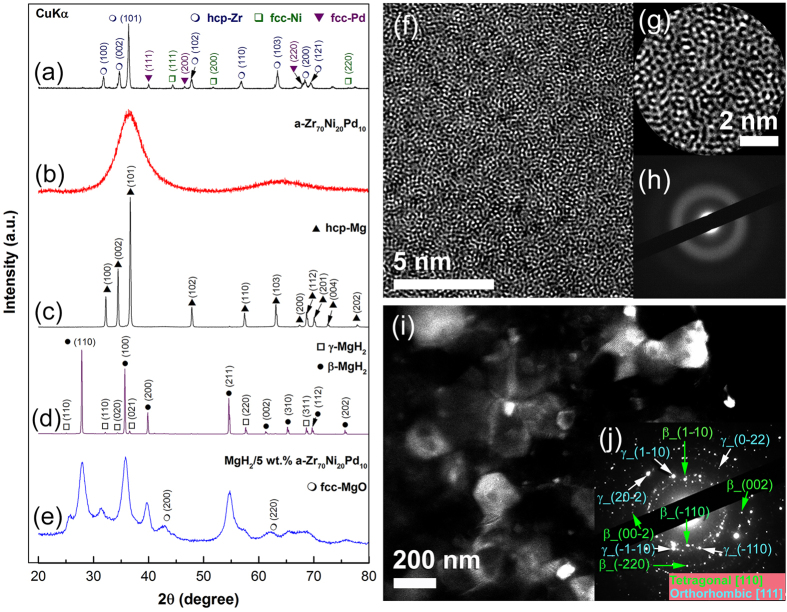
XRD patterns and FE-TEM images for selected samples obtained after different stages of ball milling time. XRD patterns of (**a**) starting elemental metallic powders of hcp-Zr, fcc-Ni and fcc-Pd, and (**b**) amorphous-Zr_70_Ni_20_pd_10_ powders obtained upon ball milling a mixture of Zr/Ni/Pd powders for 25 h, using a high-energy cryo-milling operated under a flow of liquid nitrogen. The XRD patterns of as-received hcp-Mg before starting RBM and the end-product of MgH_2_ obtained after reactive ball milling (RBM) of Mg powders for 6 h under 50 bar of a hydrogen gas pressure are shown in (**c,d**), respectively. XRD pattern of MgH_2_ powders doped with 5 wt.% of amorphous-Zr_70_Ni_20_pd_10_ and then ball milled under 50 bar of a hydrogen gas pressure for 50 h is displayed in (**e**). HRTEM image of amorphous-Zr_70_Ni_20_pd_10_ powders obtained after 50 h of cryo-milling is shown in (**f**) together with an atomic resolution TEM image (**g**) and the correspond nanobeam diffraction pattern (NBDP), (**h**). DFI image and corresponding indexed SADP of as-synthesized MgH_2_ powders obtained after 6 h of RBM time are shown in (**i,j**), respectively.

Figure 1(a) shows the X-ray diffraction (XRD) pattern of material powders at the initial stage of ball milling (0 h). The powders composed of polycrystalline mixture of hcp-Zr (70 at.%), fcc-Ni (20 at.%), and fcc-Pd (10 at.%) with large particle size, as indicated by the sharp Bragg-peaks displayed in Fig. 1(a). When the mixed powders subjected to a continuous ball milling for 25 h at an argon gas atmosphere, using a high-energy cryo-mill operated under a flow of liquid nitrogen, all of these elemental sharp Bragg peaks were disappeared and replaced by a halo-diffuse pattern of an amorphous phase, as shown in Fig. 1(b). We should emphases that milling the powders under liquid nitrogen is a necessary step used to overcome the expected agglomeration of the powders. Moreover, cryo-milling can also overcome the stacking of the powders on the milling tools that always lead to the formation of a heterogeneous glassy alloy. The cryo-ball milling process is shown in a video clip in the Supplementary Material-1-Vidio.

The image of a high-resolution transmission electron microscope (HRTEM) of the powders obtained after ball milling for 25 h (Fig. 1(f)) shows a maze-like structure without indication to the precipitation of unprocessed crystals of the starting materials (Fig. 1(g)), suggesting the formation of a single amorphous phase. The nanobeam diffraction pattern (NBDP) taken from the center of the image shows a clear halo-diffraction pattern, typically to an amorphous phase, as elucidated in Fig. 1(h). The final-product of the amorphous alloys powder obtained after 25 h of milling has a homogeneous surface structure with random close packed, as indicated by a high-resolution field emission scanning transmission electron microscope (FE-STEM) shown in the Supplementary Material-2(a). Moreover, the HRTEM images taken at different magnifications (Supplementary Material-2(b–d)) and their corresponding NBDPs (Supplementary Material-2(c–e)) suggests the absent of any intermediate crystalline phases. The as prepared amorphous Zr_70_Ni_20_Pd_10_ alloy has homogeneous chemical structure without compositional fluctuations beyond the atomic level, as elucidated by the STEM image and the corresponding energy-dispersive X-ray spectroscopy (EDS) mapping for the alloying elements of Zr, Ni, and Pd (Supplementary Material-2(f–i)).

**Figure 2:**
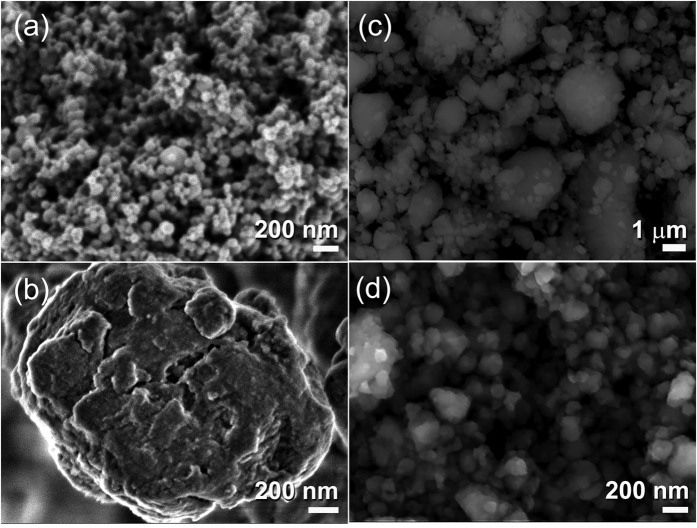
Morphological characterization of the ball milled powders obtained after differing milling time. FE-SEM micrographs of (**a**) the end-product of amorphous Zr_70_Ni_20_Pd_10_ powders obtained after 25 h of cryo-milling, and (**b**) aggregated MgH_2_ particles obtained after 6 h of RBM time. The FE-SEM micrographs of MgH_2_ doped with 5 wt.% Zr_70_Ni_20_Pd_10_ powders and them ball milled for 6 h and 50 h, are shown in (**c,d**), respectively.

The as-prepared amorphous alloy powders possess excellent morphological properties, indicated by a narrow particle size distribution laid in the range between 78–103 nm with a spherical-like morphology, as displayed in the image of field-emission scanning electron microscope (FE-SEM) displayed in Fig. 2(a). The surface area measurements, using Brunauer-Emmett Teller (BET) approach, indicated that these ultrafine amorphous nanopowder particles enjoy a high surface area of about 26.45 m^2^/g.

#### MgH_2_ powders

MgH_2_ powders were synthesized by reactive ball milling (RBM) of pure Mg powders, using a high-energy ball mill operated under 50 bar of a hydrogen gas pressure. Supplementary Material-3(a-c)) shows the photos of the vial and milling media (a) and (b) the set up performed to charge the vial with 50 bar of hydrogen gas. The photo in (c) presents the complete set up of gas-temperature-monitoring system (GST) prior to start the RBM experiment for preparing of MgH_2_ powders, using a high-energy planetary ball used in the present study. The XRD pattern of starting powders shows sharp Bragg-diffraction peaks related to hcp-Mg, as shown in Fig. 1(c). After 6 h of RBM time, a new set of Bragg-peaks was obtained, whereas all of the Bragg-lines corresponding to hcp-Mg are disappeared, indicating the formation of a new phase (Fig. 1(d)). The analysis of these new Bragg-peaks indicated the formation of polycrystalline mixture of γ-MgH_2_ and β-MgH_2_ phases with orthorhombic and tetragonal structures, respectively. After this stage of ball milling, the as-synthesized MgH_2_ powders consisted of large-grains, ranging between ~60–220 nm in diameter, with irregular morphological characterizations, as presented shown in the dark field image (DFI) presented in Fig. 1(i). The Miller-indexed selected area diffraction pattern (SADP) shown in Fig. 1(j) shows a continuous Debye-Scherrer rings related to a tetragonal phase (β-MgH_2_) overlapped with a metastable orthorhombic γ-MgH_2_ phase. At this TEM resolution, unprocessed Mg crystals could not be detected, indicating the completion of the RBM process for formation of MgH_2_ powders. The FE-SEM image for a typical aggregated MgH_2_ powder particle is shown in Fig. 2(b). The agglomerated powder, which was coated with a thick MgO resulted during SEM sample preparations (Fig. 2(b)), had an ellipsoidal-like morphology with a particle size of about 1.5 μm in diameter. The surface area of as-synthesized MgH_2_ powders after this ball milling time (6 h) was 7.30 m^2^/g.

#### MgH_2_/5 wt.% - amorphous Zr_70_Ni_30_Pd_10_ composite powders

**Figure 3:**
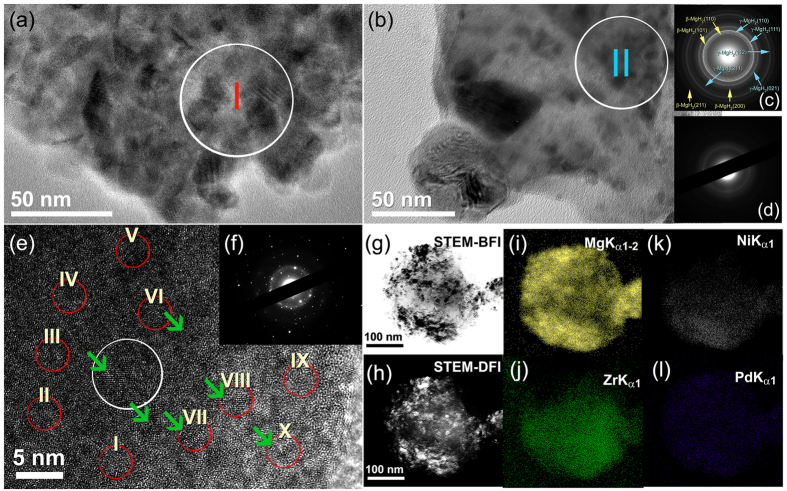
Morphological, structural, and compositional evolutions of MgH_2_/5 wt.% amorphous Zr_70_Ni_20_pd_10_ nanocomposite powders. FE-BFI images of ball milled MgH_2_/5 wt.% amorphous Zr_70_Ni_20_pd_10_ nanocomposite powders obtained after 6 h and 20 h of ball milling time are shown in (EDS analysis of the zones shown i), respectively. The corresponding SADP of the indexed zone I and zone II shown in (**a,b**), are presented in (**c,d**), respectively. The HRTEM and NBDP images for the end-product of nanocomposite MgH_2_/5 wt.% amorphous Zr_70_Ni_20_pd_10_ system obtained after 50 h of ball milling are shown in (**e,f**), respectively. The red circles shown in (**e**) refer to the zones where the X-ray-EDS analysis were achieved (Table 1). STEM-BFI and –DFI images for the end-product nanocomposite powders (50 h of ball milling) are displated in (**g,h**), respectively. The X-ray-elemental mapping for Mg, Zr, Ni and Pd corresponding to (**g**) are presented in (**i–l**), respectively.

In order to study the effect of amorphous Zr_70_Ni_30_Pd_10_ powders on improving the hydrogenation/dehydrogenation behaviors of MgH_2_ powders, the as-synthesized metal hydrides were doped with the synthetic amorphous alloy powders and high-energy ball milled for different RBM time under 50 bar of hydrogen gas pressure. A the early stage of ball milling (6 h) the composite powders consisted of heterogeneous structure of micro-scaled powders of MgH_2_, and nano-scaled amorphous powders, as elucidated in Fig. 2(c). It is worth mentioning that at this stage of milling, the powder’s chemical composition widely varied from particle to particle and within an individual particle. The field-emission bright field image (FE-BFI) of the MgH_2_-rich particle obtained after 6 h of milling is shown in Fig. 3(a). These selected aggregated particles were mainly composed of MgH_2_ grains (~35 nm in diameter), as suggested by the SADP (Fig. 3(c)) taken from region I shown in Fig. 3(a)). The composite powders obtained after 20 h showed a different feature, as elucidated in Fig. 3(b). The featureless fine structure of the amorphous powders, which have a very high surface area, became a metallic host matrix wherein the MgH_2_ were embedded to form a typical composite powders, as shown in Fig. 3(b). The SADP corresponding to zone II (Fig. 3(b) shows an overlap between the two MgH_2_ phases (tetragonal (β) and orthorhombic (γ)) and the halo-amorphous pattern displayed in 3(d). The SADP displayed in 3(d) shows a continuous Debye-ring diffraction pattern of β-and γ-MgH_2_ phases with the absence of sharp spots. This implies the formation of a nanocrystalline MgH_2_ grains embedded into the amorphous matrix. However, the “guest phase” of MgH_2_ grains that became somewhat finer (as a result of further milling) in sizes (ranging between 8–32 nm), were still heterogeneously distributed into the metallic matrix, as indicated by the dark regions presented in Fig. 3(b).

In order to ensure the homogeneous distribution of MgH_2_ grains into the amorphous matrix, the composite powders were furtherly ball milled for 50 h. The XRD pattern of nanocrystalline-MgH_2_/5 wt.% amorphous Zr_70_Ni_30_Pd_10_ composite powders obtained after the end of processing time (50 h) is presented in Fig. 1(e). The primary- and secondary haloes become very broad without indication to the existence of medium- or long-range ordered structure, as shown in Fig. 1(e). Moreover, the Bragg-diffraction peaks related to MgH_2_ (γ and β phases) show significant broadening (Fig. 1(e)), indicating the effect of RBM time on grain refining and formation of nanocrystallites. Those Bragg peaks shown in Fig. 1(e), which are related to fcc-MgO phase, came from the oxidation of the powder surfaces during preparation the XRD sample outside the helium-atmosphere glove box. The nanocomposite powders of this end-product comprised of ultrafine particles, laid in the range between 50 nm up 420 nm in diameter (Fig. 2(d)) and having a surface area of 16.80 m^2^/g.

The HRTEM image taken near the edge of MgH_2_/5 wt.% amorphous Zr_70_Ni_30_Pd_10_ composite particle obtained after 50 h of RBM time is shown in Fig. 3(e) together with the corresponding NBDP (Fig. 3(f)). Overall, the composite powders obtained after this stage of milling consisted of continuous amorphous matrix (maze-like morphology shown in Fig. 3(e)) hosting ultrafine nanoclusters (~4 nm in diameter) of order-structure (related to MgH_2_), as indexed by the green arrows labels shown in Fig. 3(e). It is worth mentioning that the MgH_2_ grains were distributed into the metal amorphous matrix in a segregation fashion with the absence of agglomerates or aggregated grains, as displayed in Fig. 3(e). The NBDP (Fig. 3(f)) corresponding to the white circular lable shown in Fig. 3(e) shows halo-diffraction pattern related to amorphous Zr_70_Ni_30_Pd_10_ coexisted with spot-like pattern came from nanocrystalline γ- and β-MgH_2_ phases oriented in different axial directions.

In order to investigate the elemental chemical composition of the nanocomposite powders obtained after 50 h of ball milling, the powders were subjected to intensive local EDS analysis, using a beam focus of 5 nm. The red-circular zones labeled by Roman numerals (I to X) in Fig. 3(e) refer to the EDS selected zones for performing the compositional analysis. Table 1 shows the detailed of these analyses in weight percent (wt.%). From these analyses, one can say that the composition of the nanocomposite powders varying from 72.6 to 99.2 wt.% Mg. This corresponding to 0.8 to 27.4 wt.% Zr_70_Ni_30_Pd_10_. More information about the distribution of MgH_2_ in the amorphous matrix composite EDS mapping approach was performed. The STEM-BFI and the corresponding STEM-DFI of a selected nanocomposite MgH_2_/5 wt.% - amorphous Zr_70_Ni_30_Pd_10_ powder particle are shown in Fig. 3(g,h), respectively. The powder has a nearly spherical morphology (Fig. 3(g)) and containing fine lenses of less than 10 nm in diameter homogeneously distributed into the fine structured powder particle, as shown in Fig. 3(h). The fine spherical lenses were corresponding to Mg (Fig. 3(i)), whereas the elemental composition of the particle related to the elemental Zr, Ni and Pd, as indicated in the elemental mapping presented in Fig. 3(j–l), respectively.

### Thermal stability

**Figure 4:**
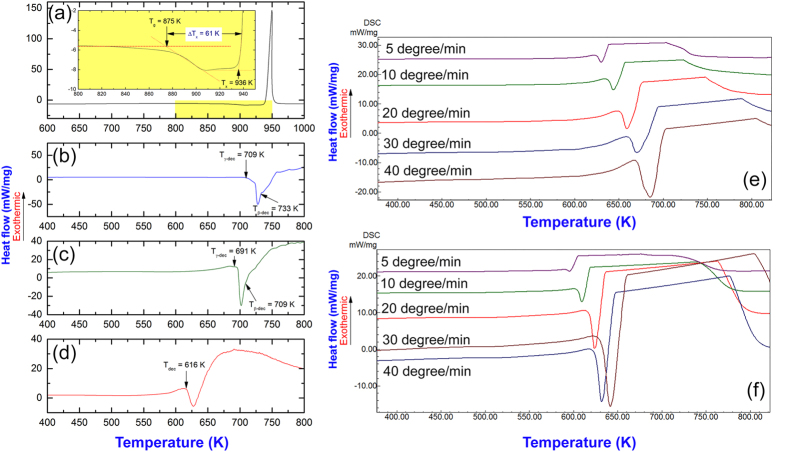
Thermal analysis of Zr_70_Ni_20_pd_10_ metallic glassy powders and corresponding MgH_2_/5 wt.% metallic glassy nanocomposite powders. The dependence of decomposition temperature on the metallic glassy additives and ball milling time are elucidated. DSC curves of (**a**) metallic glassy Zr_70_Ni_20_pd_10_ powders obtained after 25 h of cryo- milling time, and (**b**) MgH_2_ powders after RBM time for 6 h. The DSC curves of MgH_2_ powders doped with 5 wt.% of metallic glass Zr_70_Ni_20_pd_10_ powders and then ball milled under 50 bar of a hydrogen gas pressure for 10 h and 50 h are shown in (**c,d**), respectively. The DSC curves achieved at different heating rates for MgH_2_ powders obtained after 6 h of RBM time and after ball milling with 5 wt.% of metallic glass Zr_70_Ni_20_pd_10_ powders for 50 h are presented in (**e,f**), respectively.

Differential scanning calorimetry (DSC) performed at a heating rate of 20 °C under a helium gas flow of 75 ml/min was employed in order to investigate the crystallization properties of the amorphous matrix powders and the decomposition behavior of MgH_2_ powders. Figure 4(a) displays the DSC curve of amorphous Zr_70_Ni_30_Pd_10_ powders obtained after 25 h of RBM time. The DSC scan reveals two reaction events taken place at onset temperature of 875 K (shown in a different scale inset of the figure) and 936 K, as shown in Fig. 4(a). The first event is an endothermic reaction refers to the glass transition temperature (T_g_) related to the form of glassy phase, whereas the second event takes place through a sharp exothermic peak related to the crystallization of the metallic glassy phase, as shown in Fig. 4(a). The differences between the crystallization temperature (T_x_) and T_g_ refers to the supercooled liquid region (ΔT_x_; T_x_-T_g_ = 61 K).

The DSC scan of as-synthesized MgH_2_ powders obtained after 6 h of RBM (before mixing with the metallic glassy powders) reveals shoulder-like endothermic peaks, as shown in Fig. 4(b). The metastable phase of γ-MgH_2_ tends to decompose at lower temperature (Tγ_-dec_ = 709 K), when compared with the decomposition temperature of β-MgH_2_ (Tβ_-dec_ = 733 K), as displayed in Fig. 4(b). Both Tγ_-dec_ and Tβ_-dec_ shifted to the low temperature side upon ball milling with metallic glassy Zr_70_Ni_30_Pd_10_ powders for 10 h, and recorded to be 691 K and 709 K, respectively (Fig. 4(c)). Significant decreasing in the decomposition temperature (616 K) for MgH_2_ is realized of the sample mixed with 5 wt.% Zr_70_Ni_30_Pd_10_ powders and milled for 50 h, as displayed in Fig. 4(d). This implies the outstanding effect of metallic glassy and the RBM time for destabilizing the MgH_2_ phase.

The activation energy for MgH_2_ powders obtained after 6 h of RBM time and nanocomposite MgH_2_/5 wt.% amorphous Zr_70_Ni_30_Pd_10_ powders obtained after 50 h of the ball milling time were investigated by DSC analysis conducted with different heating rates (k) of 5, 10, 20, 30, and 40 °C/min and shown in Fig. 4(c,d), respectively. All the scans for both material powders revealed single endothermic events related to the decomposition of MgH_2_. While the peak height increased proportionally with the increasing the heating rates, the peak temperatures (T_p_) were significantly shifted to the higher temperature side upon increasing the heating rates from 5 °C/min to 40 °C/min, as shown in Fig. 4(c,d).

In the present work, the activation energy (E_a_) of dehydrogenation of pure MgH_2_ and nanocomposite MgH_2_/5 wt.% amorphous Zr_70_Ni_30_Pd_10_ powders was calculated according to the Arrhenius equation:


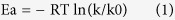


where k is a temperature-dependent reaction rate constant, R is the gas constant, and T is the absolute temperature. The E_a_ values were determined by measuring the decomposition the T_p_ corresponded to the different heating rates (k) and then plotting ln(k) versus 1/T_p_. The Ea values were obtained from the slope of line (-E/R, where R is the gas constant). Based on these measurements, MgH_2_ powders obtained after 6 h of RBM time showed a high E_a_ value (143 kJ/mol), indicating a high stability against decomposition. In contrast, E_a_ of nanocomposite MgH_2_/5 wt.% amorphous Zr_70_Ni_30_Pd_10_ powders obtained after 50 h of ball milling showing a lower value (92 kJ/mol), indicating a significant destabilization of the MgH_2_ upon high-energy ball milling with amorphous phase. However, the apparent E_a_ of our system is less than those values for pure MgH_2_ and Mg_85_In_5_Al_5_Ti_5_ (125.2 kJ/mol)^42^ systems, but it is well above the reported value for MgH_2_ powders coated by Ti-based thin films (30.8 kJ/mol)^43^ and CeH_2.73_-MgH_2_-Ni nanocomposite (63 kJ/mol)^39^.

### Hydrogenation/dehydrogenation behaviors

#### Pressure-Composition-Temperature

**Figure 5:**
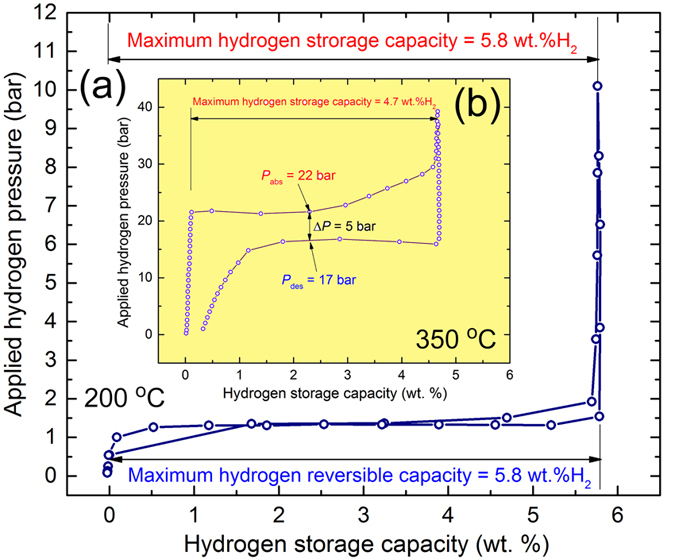
Effect of metallic glassy additives and milling time on the hydrogenation/dehydrogenation behavior of MgH_2_ powders. The PCT curves for the end-products of ball-milled MgH_2_/5 wt.% metallic glass Zr_70_Ni_20_pd_10_ nanocomposite and MgH_2_ powders are shown in (**a,b**), respectively. Whereas the PCT experiment conducted at 200 °C for the nanocomposite powders, it was accomplished at 350 °C for MgH_2_ system.

Figure 5(a,b) shows the pressure-composition-temperature (PCT) curves investigated at 200 °C 350 °C for nanocomposite MgH_2_/5 wt.% amorphous Zr_70_Ni_30_Pd_10_ powders obtained after 40 h of ball milling (Fig. 5(a)) and MgH_2_ powders obtained after 6 h of RBM time (Fig. 5(b)), respectively. For successful hydride formation (complete absorption) of MgH_2_ sample, high temperature (350 °C) and pressure (P_abs_, in the range between 200 mbar to 40 bar) were required to absorb about 4.7 wt.% H_2_, as shown in Fig. 5(b). This indicates that the sample requires the application of higher temperature to get its theoretical hydrogen storage capacity (7.6 wt.% H_2_). Moreover, the sample showed poor dehydrogenation behavior, indexed by exhibiting a clear pressure hysteresis with significant large gabs (~17 bar) between the pressure needed for absorption (hydride formation), P_abs_ and the pressure required for hydride decomposition, P_des_, as presented in Fig. 5(b). In addition, pure MgH_2_ powders obtained after 6 h of RBM time failed to desorb its stored hydrogen content completely even after 12 h of the desorption time (Fig. 5(b)).

In contrast to the MgH_2_ sample, the nanocomposite powders showed an excellent PCT hydrogenation/dehydrogenation curves, indexed by the complete hydrogen sorption/desorption event at lower temperature (200 °C) and pressure (50 mbar to 10 bar), as shown in Fig. 5(a). At such relative low temperature and pressure, the nanocomposite sample reached to a higher value of hydrogen storage capacity (5.8 wt.%) when compared with pure MgH_2_ (4.7 wt.% H_2_). Moreover, the plateau region for the nanocomposite sample was very flat with negligible slope with the absence of multistep hydrogenation/dehydrogenation, as shown in Fig. 5(a). In addition, the sample succeed to achieve complete desorption through an almost flat PCT curve with minimal difference value (~72 mbar) between P_abs_ and P_des_, as shown in Fig. 5(a). Moreover, our system possessed rather low-pressure plateau (~1.5 bar/200 °C) closed to that value reported for Mg_3_PrNi_0.1_ (1.6 bar/297 °C)^38^ Mg_80_Ce_18_Ni_2_ (3 bar/311 °C)^39^, but it shows lower pressure plateau when compared with mechanically alloyed Mg-5 at.%In (8 bar/350 °C)^40^, Mg-5at.%Sn revealed (~5 bar/323 °C)^41^, and Mg_85_In_5_Al_5_Ti_5_ (7 bar/380 °C)^42^. The hydrogen storage capacity of MgH_2_/5 wt.% amorphous Zr_70_Ni_30_Pd_10_ system possessed high hydrogen storage capacity (5.8 wt.%), being closed to that one (~5.5 wt.%) reported for both Mg(In) solid solution^40^ and Mg–Sn nanocomposite^41^. This storage capacity of our system is far above other MgH_2_-based hydrides, such as Mg_3_PrNi_0.1_ (2.5 wt.%)^38^, Mg_80_Ce_18_Ni_2_ (3.5 wt.%)^39^, however, it is a bit below than the storage capacity reported for MgH_2_ catalysed by Ti-based nanocoating (6.05 wt.%)^43^.

#### Kinetics of absorption

**Figure 6:**
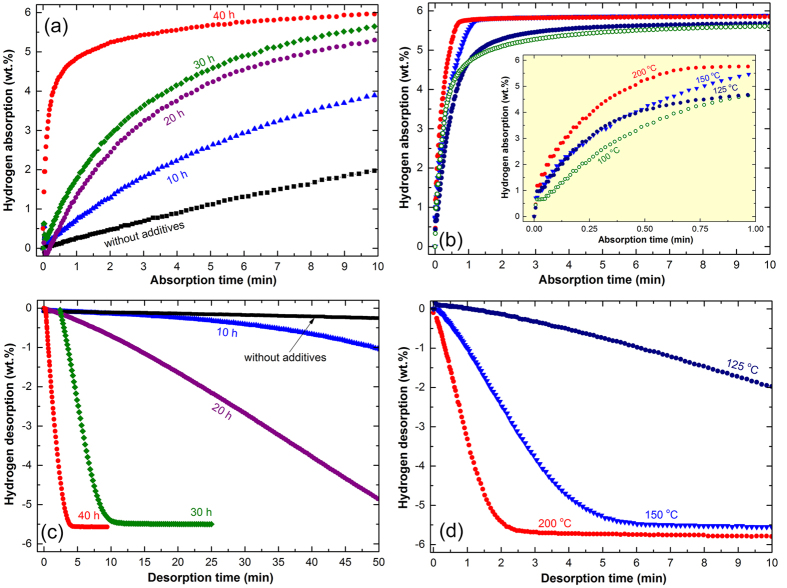
Hydrogenation/dehydrogenation kinetics of MgH_2_ as a function of applied temperature, metallic glassy additives, and ball milling time. Dependence of the absorption (**a**) and desorption (**c**) kinetics of MgH_2_, measured at 175 °C on the milling time with metallic glassy Zr_70_Ni_20_pd_10_ powders. The absorption and desorption kinetics examined at different temperatures for composite MgH_2_/5 wt.% amorphous-Zr_70_Ni_20_pd_10_ powders obtained after 50 h of milling time are shown in (**b,d**), respectively.

One major problem restricting the potential applications of MgH_2_ compound in real fuel cell and energy storage applications is its very slow hydrogen uptake/release kinetics that required the application of a high temperature (above 400 °C) to be enhanced. For example, at 175 °C, the as-synthesized MgH_2_ powders obtained after 6 h of RBM (without additives of metallic glassy powders) require 10 min to absorb about 2 wt.% H_2_, as shown in Fig. 6(a). When MgH_2_ mixed with the metallic glassy powders and milled for 10 h, a remarkable improvement on the absorption kinetics is achieved, indexed by an increase in the hydrogen amount absorbed within 10 min to 3.89 wt.%, as shown in Fig. 6(a). Increasing the RBM time led to enhance the absorption kinetics, indexed by the absorbed amount hydrogen recorded for the samples obtained after 20 h (5.26 wt.%), and 30 h(5.68 wt.%). As elucidated in Fig. 6(a). The sample obtained after ball milling with the metallic glassy powders for 40 h shows outstanding hydrogenation kinetics, indicated by the very short time (~2 min) required to absorb ~5 wt.%H_2_, as shown in Fig. 6(a). This sample reaches to its maximum storage capacity (~5.8 wt.%H_2_) after only 9 min, as presented in Fig. 6(a).

Figure 6(b) shows the temperature effect, ranging between 100–200 °C on the absorption kinetics of composite MgH_2_/5 wt.% metallic glassy Zr_70_Ni_30_Pd_10_ powders obtained after 50 h of RBM time. The relation between the absorbed hydrogen during the first minute of the experiment is shown inset of Fig. 6(b). The fabricated composite powders reveal excellent hydrogenation characteristics, indexed by their high capability of absorbing hydrogen (~4.6 wt.%) within a short time (1 min) at low temperature (100–125 °C), as displayed inset of Fig. 6(b). They reached together to their saturation values of 5.6 wt.% H_2_ after 6.5 min, as presented in Fig. 6(b). Increasing the applied temperature to 150 °C improves the absorption kinetics, as suggested by the higher hydrogen absorbed (~5.5 wt.%) in 1 min (inset Fig. 6(b)). This sample reached to its saturated capacity value of 5.8 wt.% H_2_ after about 1.18 min, as displayed in Fig. 6(b). Significant improvement is achieved at 200 °C when the sample reached to a hydrogen capacity of 5.8 wt.% within 0.75 min (inset Fig. 6(b)), and does not show any degradation upon increasing the absorption time to 10 min, as shown in Fig. 6(b).

#### Kinetics of desorption

Figure 6(c) shows the dependence of desorption kinetics for MgH_2_ measured at 175 °C on the metallic glassy additive and RBM time. Originally, pure MgH_2_ powders obtained after 6 h of RBM time has a poor desorption kinetics, indexed by the low value of hydrogen released(~0.35 wt/%) after 50 min of desorption time (Fig. 6(c)). When MgH_2_ mixed with the metallic glassy the powders and milled for 20 h, a better desorption kinetics can be attained, as indicated by a higher H_2_ desorbed value (~1 wt.%) obtained after 50 min (Fig. 6(c)). Remarkable improving on the desorbed kinetics is realized for the composite sample obtained after 20 h (4.88 wt.% H_2_/50 min) and 30 h (5.6 wt.% H_2_/26 min), as shown in Fig. 6(c). The composite sample powders obtained after 40 h of RBM time, shows excellent dehydrogenation characteristics, indexed by the very short time (4.6 min) required to release about 5.7 wt.%H_2_, as displayed in Fig. 6(c).

The temperature effect on the desorption kinetics for the sample obtained after 50 h of RBM time is shown in Fig. 6(d). The composite powders examined at 125 °C desorbed about 2 wt.% H_2_ within 10 min, as shown In Fig. 6(d). Increasing the applied temperature to 150 °C leads to enhance the dehydrogenation kinetic behavior, indicated by the shorter time (6.5 min) to release about 5.5 wt.% H_2_. This value is saturated at 5.6 wt.% H_2_ after 9.35 min, as shown in Fig. 6(d). Outstanding enhancement for the desorption kinetics is attended of the sample measured at 200 °C, showing a very short time (3.8 min) needed to release about 5.7 wt.%H_2_ (Fig. 6(d)).

### Cycle-life-time

The behavior of MgH_2_/5 wt.% metallic glassy Zr_70_Ni_30_Pd_10_ powders obtained after 50 h of RBM time upon subjecting to continuous hydrogenation/dehydrogenation repetitions for 100 times was studied at 200 °C under a hydrogen gas pressure ranging between 200 mbar to 10 bar. The powders were firstly activated by applying cyclic hydrogen gas sorption/desorption under pressure of 35 bar at 325 °C for 20 continuous cycles. This treatment is necessary for surface cleaning of the powders and to break down the oxide phase for the surface.

**Figure 7:**
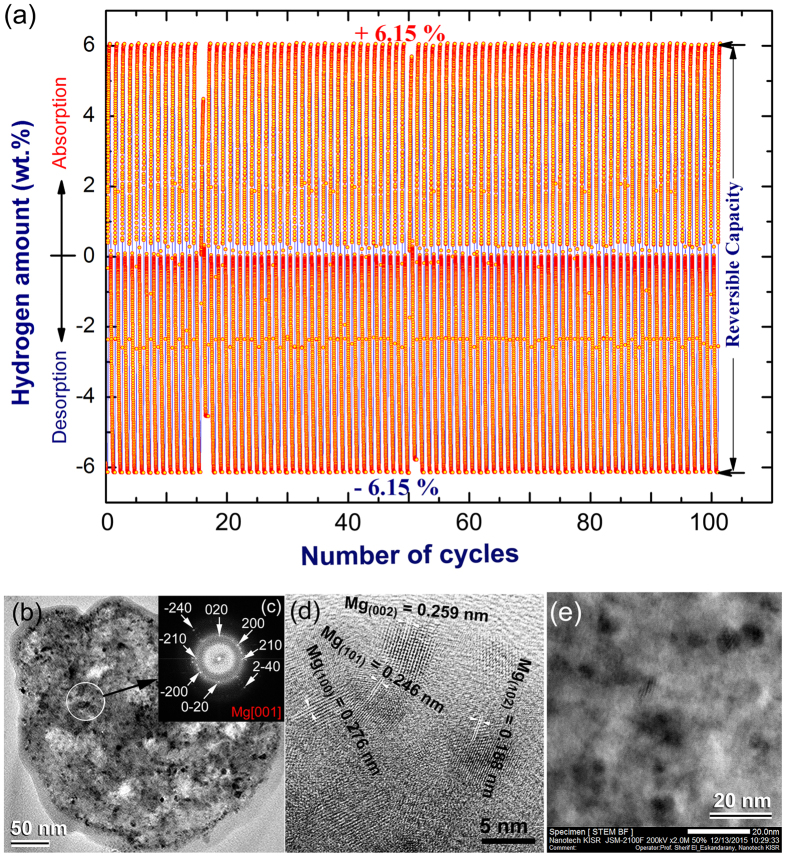
Cycle-life-time conducted for the end-product of nanocomposite powders and the corresponding morphological evolutions after completion the test. (**a**) Successful completed 100-hydrogenation/dehydrogenation cycles performed at 200 °C under a hydrogen gas pressure ranging between 200 mbar–10 bar for composite MgH_2_/5 wt.% amorphous-Zr_70_Ni_20_pd_10_ powders obtained after 50 h of milling time. The BFI and SADP of the sample taken after completion of 100 cycles are presented together with the SADP in (**b,c**), respectively. The HRTEM image and high-magnification STEM-BFI are shown in (**d,e**), respectively.

Figure 7(a) shows the hydrogen absorbed/desorbed cycles achieved continuously for 100 times at a temperature of 200 °C. It should be emphasized that surface treatment of the powders led to improve its capability of hydrogen absorption, reached to 6.15 wt.%, as shown in Fig. 7(a). No remarkable degradation in the hydrogen storage capacity could be detected even after 100 cycles, as shown in Fig. 7(a). The kinetics of hydrogenation/dehydrogenation remaining constant with nearly constant absorption and desorption values of 6.15 wt.%.

The BFI micrograph of the sample taken after the completion of 100 sorption/desorption cycles conducted at 200 °C is shown in Fig. 7(b). The powder consisting of featureless morphology where numerous ultrafine dark-contrast spherical grains were embedded into the light-gray fine matrix (Fig. 7(b)). It should be notifying that the Mg powders were segregated and fairly distributed into the metallic glassy matrix. These Mg grains maintained their original sizes without severe grain growth even after performing the sample for 100 cycles. The Fast Fourier Transform (FFT) image of the zone indexed in Fig. 7(a) shows halo diffraction rings of an amorphous phase coexisted with diffracted spots corresponding to hcp-Mg crystal oriented to [001], as shown in Fig. 7(c). The HRTEM image of selected Mg grains are shown with a higher magnification in Fig. 7(d). Clear Moiré-like fringes with different interplanar spacing (d) of fine grains (~5 nm in diameter) are shown in Fig. 7(d). These displayed grains having d spacing values of 0.259 nm, 0.188 nm, 0.276 nm and 0.246 nm that match well with the (002), (102), (100) and (101) lattice indexes of hcp-Mg, respectively. The absence of severe grain growth in the Mg grains after completion of cyclic test can be attributed to the surrounded hard metallic glassy matrix that played the role of a grain growth inhibitor. Fig. 7(e) shows a high-resolution STEM-BFI displaying the host metallic glassy matrix and spherical metallic Mg grains. The matrix maintained its fine structure without any evidences of crystallizations or formation of medium-range ordered phase during the hydrogenation/dehydrogenation cycles. The thermal stability of our prepared metallic glassy phase used in the present study and the absence of phase transformations during the hydrogenation/dehydrogenation cycles leads to a sustainable hydrogen storage capacity with constant hydrogen uptake/releasing kinetics, as shown in Fig. 7(a).

## Discussions

In contrast to the traditional catalyst families (e.g. elemental metals, metal alloys, compounds) used to improve the kinetic behaviors of MgH_2_ powders, the present study proposes a metastable metallic glassy Zr_70_Ni_20_Pd_10_ alloy nanopowder as a superior enhancer leading to modify the hydrogenation/dehydrogenation properties of MgH_2_. This prepared metallic glassy phase is homogeneous in structure and uniform in composition beyond the atomic level (Fig. 1(f) Supplementary Fig. S2). Moreover, our fabricated metallic glassy nanopowder possesses a high thermal stability, indicated by the large ΔT_x_ (61 K) and high T_x_ (936 K) values, as shown in Fig. 4(a). Thus, Zr_70_Ni_20_Pd_10_ metallic glassy powders did not undergo to any structural changes upon processing MgH_2_/5 wt.% Zr_70_Ni_20_Pd_10_ nanocomposite powders at relatively lower temperature of less than 800 K (527 °C).

During the early stage (<10 h) of ball milling a mixture of MgH_2_ and 5wt.% Zr_70_Ni_20_Pd_10_ metallic glassy powders, the ball-powder-ball collusions (Supplementary Fig. S4) led to break down the large MgH_2_ powder particles (Supplementary Fig. S5(a,b)) and assisted adherence of the glassy fine powders onto the surface of MgH_2_ particles (Supplementary Fig. S5(c)). During this stage of milling, the hard metallic glassy nanopowders penetrated the oxide layer formed on the surfaces of MgH_2_ powders to create micro-holes on their surfaces (Supplementary Fig. S5(d,e)).

Increasing the ball milling time (10 to 20 h) led to “migrate” a large volume fraction of metallic glassy powders through the cavities and micro-channels created in the body of MgH_2_ particles and located their grains and at the grain boundary zones (Supplementary Fig. S5(f)). Such glassy powders, which acted as “micro-grain splitter” led to break up the large MgH_2_ grains along their weak grain boundary zones (Supplementary Fig. S5(g)) and forming finer grains (Supplementary Fig. S5(h)). Further ball milling time (20–40 h) resulting an increase the volume fractions of MgH_2_ fine grains and the number of grain boundaries (Supplementary Fig. S5(h)). Since the hydrogen diffusion is much faster along the grain boundaries when compared with inside grains, the hydrogenation/dehydrogenation kinetic behaviors of MgH_2_ were gradually improved (Fig. 6(a,c)) with increasing the number of “liberated” grains (Supplementary Fig. S5(i)).

During the last stage of ball milling (40–50 h), the role of ball-powder-ball collusions on achieving further refining of MgH_2_ powder particles was almost absent since the size of the powders became ultrafine (less than 1 μm)^8^. Accordingly, the final refining process was attained by the nanosized metallic glassy powders, which were homogeneously distributed within MgH_2_ to form typical homogeneous nanocomposite powders (Supplementary Fig. S5(j–l)). The end-product of the nanocomposite powders obtained after 50 h (Supplementary Fig. 5(m)) possessed excellent morphological characteristics, indexed by a homogeneous dispersions of equal nano-sized MgH_2_ grains segregated into the metallic glassy powders, without agglomeration. This nanocomposite structured powders facilitated fast hydrogen diffusion, as suggested by Jeon for MgH_2_ NCs/PMMA nanocomposite system^35^. During the particle/grain, refining “long-voyage” extended to 50 h of high-energy ball milling, MgH_2_ powders were subjected to continuous mechanical deformations created by the impact and shear forces generated from milling, and micro-abrasive milling media of the balls and metallic glassy powders, respectively. These forces were translated into sever plastic deformation, and lattice imperfections created into the MgH_2_ powders (Supplementary Fig. S5(n)).

Based on the results of the present study, high surface area (26.45 m^2^/g) of metallic glassy Zr_70_Ni_20_Pd_10_ nano-spherical powders played a superior role for improving both of hydrogenation/dehydrogenation kinetics of MgH_2_ powders. The PCT curve of the formed nanocomposite powder in the present work, containing 10wt.% Pd did not multi-steps process suggested by Yamada *et al*. for Mg-Pd, Mg-Nd, and Mg-Pd-Nd systems^37^.

When our results are compared with the In the present work, the formation of intermediate phases of Mg_2_NiH_6_ and Mg_6_Pd upon hydrogenation/dehydrogenation process in temperatures ranging between 125–250 °C could not be detected. This can suggest the absent of the common catalytic role of the metallic glassy powders when mixed with MgH_2_ powders. Thus, it can be concluded that the kinetics of MgH_2_ powders were enhanced by a drastic grain refining and the formation of large volume fractions of segregated grains that facilitated fast hydrogen diffusion along their numerous number of grain boundaries. This grain refinement was achieved upon using abrasive metallic glassy powders.

In summary, the hydrogenation/dehydrogenation kinetics of MgH_2_ powders prepared by reactive ball milling technique was greatly improved upon mechanically-induced doping with a small mole fraction (5 wt.%) of Zr_70_Ni_20_Pd_10_ metallic glassy powders. Adding such a metallic metastable phase led to destabilize the MgH_2_ and improved its kinetics. The as-synthesized nanocomposite MgH_2_/5 wt.% Zr_70_Ni_20_Pd_10_ powders possessed high density of hydrogen and exhibited irreversible hydrogenation/dehydrogenation process taking place at low pressure and temperature with a very short time.

## Methods

### Preparation of the metallic glassy powders

Pure Zr (100 μm, 99% purity), Ni (10 μm, 99.9% purity) and Pd (10 μm, 99.5% purity) metal powders provided by Alfa Aesar - USA, were used as starting alloying elements. The powders were balanced and manually mixed inside a helium (He) gas atmosphere (99.99%)-glove box (UNILAB Pro Glove Box Workstation, mBRAUN, Germany) to give the starting charge (1 g) with an average nominal composition of Zr_70_Ni_20_Pd_10_. The powders were then sealed together with five Cr- stainless steel balls (10 mm in diameter) into a FeCr steel vial (20 ml in volume, Retsch, Germany), using a ball-to-powder weight ratio as 50:1. In order to avoid the agglomeration of the metallic powders during the ball milling process, a cryo-mill system provided by Retsch was used. In this experiment, the vial containing the balls and powders was mounted on the cryo-milling system where the process taking place under continuous cooling, using liquid nitrogen flow. The liquid nitrogen circulated through the system and was continually replenished from an auto fill system in the exact amount, which is required to keep the temperature at −196 °C. This milling process was carried out with a frequency of 25 Hz for 25 h. The end-product obtained after 25 h was discharged in the He-atmosphere glove box.

### Preparation of the metal hydride powders

Elemental Mg metal powders (~80 μm, 99.8% provided by Alfa Aesar - USA), and hydrogen gas (99.999%) were used as starting materials. An amount of 5 g Mg was balanced inside a He gas atmosphere (99.99%) - glove box (UNILAB Pro Glove Box Workstation, mBRAUN, Germany). The powders were then sealed together with twenty five hardened steel balls into a hardened steel vial (150 ml in volume), using a gas-temperature-monitoring system (GST; supplied by evico magnetic, Germany). The ball-to-powder weight ratio was 40:1. The vial was then evacuated to the level of 10^−3^ bar before introducing H_2_ gas to fill the vial with a pressure of 50 bar. The reactive ball milling (RBM) process was carried out at room temperature, using a high energy ball mill (Planetary Mono Mill PULVERISETTE 6, Fritsch, Germany). After 6 h of RBM time, the powders were discharged from the vial inside the glove box and sealed in Pyrex vails. The as-synthesized MgH_2_ powders were then mixed in the glove with the desired weight percentage (5%) of Zr_70_Ni_20_Pd_10_ amorphous powders, using an agate mortar and pestle. The mixed powders were charged together with twenty five hardened steel balls into the vial and sealed under He gas atmosphere. The vial was then filled with 50 bar of hydrogen gas atmosphere and mounted on the high-energy ball mill. The milling process was interrupted after selected time (10, 20, 30, 40, and 50 h) and the powders obtained after an individual milling time were completely discharged into 8 Pyrex vails for different analysis. The contamination contents of Fe and Cr of the powders obtained after 50 h of ball milling were Based on the EDS analyses of at least 50–60 powder particles, the average contents of Fe and Cr introduced to the powders upon using tempered Cr-steel milling tools were 0.86 and 0.19 wt.%, respectively.

### XRD and TEM

The crystal structure of all samples was investigated by XRD with CuKα radiation, using 9kW Intelligent X-ray diffraction system, provided by SmartLab-Rigaku, Japan. The local structure of the synthesized material powders was studied by 200 kV-field emission high resolution transmission electron microscopy/scanning transmission electron microscopy (HRTEM/STEM) supplied by JEOL-2100F, Japan, and equipped with Energy-dispersive X-ray spectroscopy (EDS) supplied by Oxford Instruments, UK.

### Thermal stability

Differential scanning calorimetry (DSC)/differential thermal analysis (DTA) unit, provided by Setaram –France with a heating rate of 20 °C/min was employed to investigate the glass transition temperature, and thermal stability indexed by the crystallization temperature and enthalpy change of crystallization for the metallic glassy powders. Shimadzu Thermal Analysis System/TA-60WS, using differential scanning calorimeter (DSC) was employed to investigate the decomposition temperatures of MgH_2_-based composite powders with a heating rate of 20 °C/min. The activation energy for pure MgH_2_ and MgH_2_/5wt.% Zr_70_Ni_20_Pd_10_ was investigated, using Arrhenius approach with different heating rates (5,10,20,30,40 °C/min).

### The hydrogenation/dehydrogenation behaviors

The hydrogen absorption/desorption kinetics were investigated via Sievert’s method, using PCTPro-2000, provided by Setaram Instrumentation, France, under hydrogen gas pressure in the range between 200 mbar to 10 bar. The samples were examined at different temperatures of 100, 125, 150, 175, and 200 °C.

## Additional Information

**How to cite this article**: El-Eskandarany, M. S. Metallic glassy Zr_70_Ni_20_Pd_10_ powders for improving the hydrogenation/dehydrogenation behavior of MgH_2_. *Sci. Rep*. **6**, 26936; doi: 10.1038/srep26936 (2016).

## Supplementary Material

Supplementary Information

Supplementary Video S1

## Figures and Tables

**Table 1:** Local EDS analysis of the zones shown in Fig. 3(e) for as-fabricated MgH_2_/5wt.% Zr_70_Ni_20_pd_10_ nanocomposite powders obtained after 50 h of ball milling time.

Zone	Composition (wt.%)	
	Mg	ZrNiPd
I	72.6	27.4
II	81.3	18.7
III	84.7	15.3
IV	97.2	2.8
V	94.6	5.4
VI	93.8	6.2
VII	98.8	1.2
VIII	99.2	0.8
IX	95.1	4.9
X	97.6	2.4
