# Supplementary material for: Metallic glassy Zr70Ni20Pd10 powders for improving the hydrogenation/dehydrogenation behavior of MgH2
Source: Sci Rep. 2016 May 25;6:26936. doi: 10.1038/srep26936 (PMC4879527; doi:10.1038/srep26936)
Supplement: Supplementary Information [file srep26936-s1.doc]

**Metallic glassy Zr_70_Ni_20_Pd_10_ powders for improving the hydrogenation/dehydrogenation behaviours of MgH_2_**

M. Sherif El-Eskandarany

**Supplementary Materials**

**Supplementary Video Clip**

This clip shows the cryo-milling processing of elemental Zr, Ni, and Pd metallic powders performed under a stream of liquid nitrogen in order to avoid the powders agglomeration that usually leading to produce a heterogeneous product.


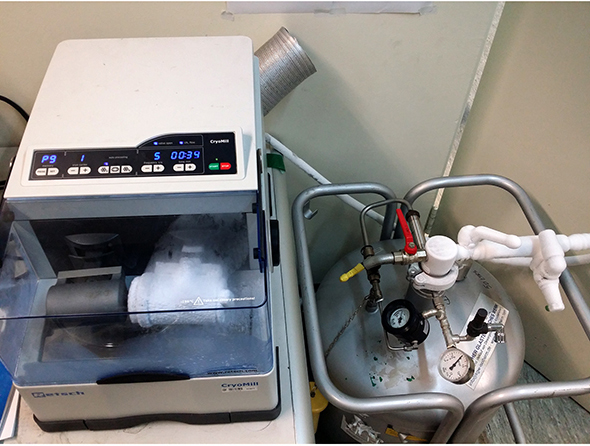


**Supplementary Figure S1**

Photo of the cryo-mill used in the present study to synthesize metallic glassy powders of Zr_70_Ni_20_pd_10_





**Supplementary Figure S2**

1. High resolution STEM-BFI taken at a magnification of 150 M times of metallic glassy Zr_70_Ni_20_pd_10_ powders obtained after 25 h of cryo-milling time. The HRTEM images of the glassy powders are shown in (b) and (d) together with their corresponding NBDPs (c, and e). The STEM-BFI image of the metallic glassy powders is shown in (f) together with the corresponding X-ray EDS elemental mapping of Zr (g), Ni (h) and Pd(i).


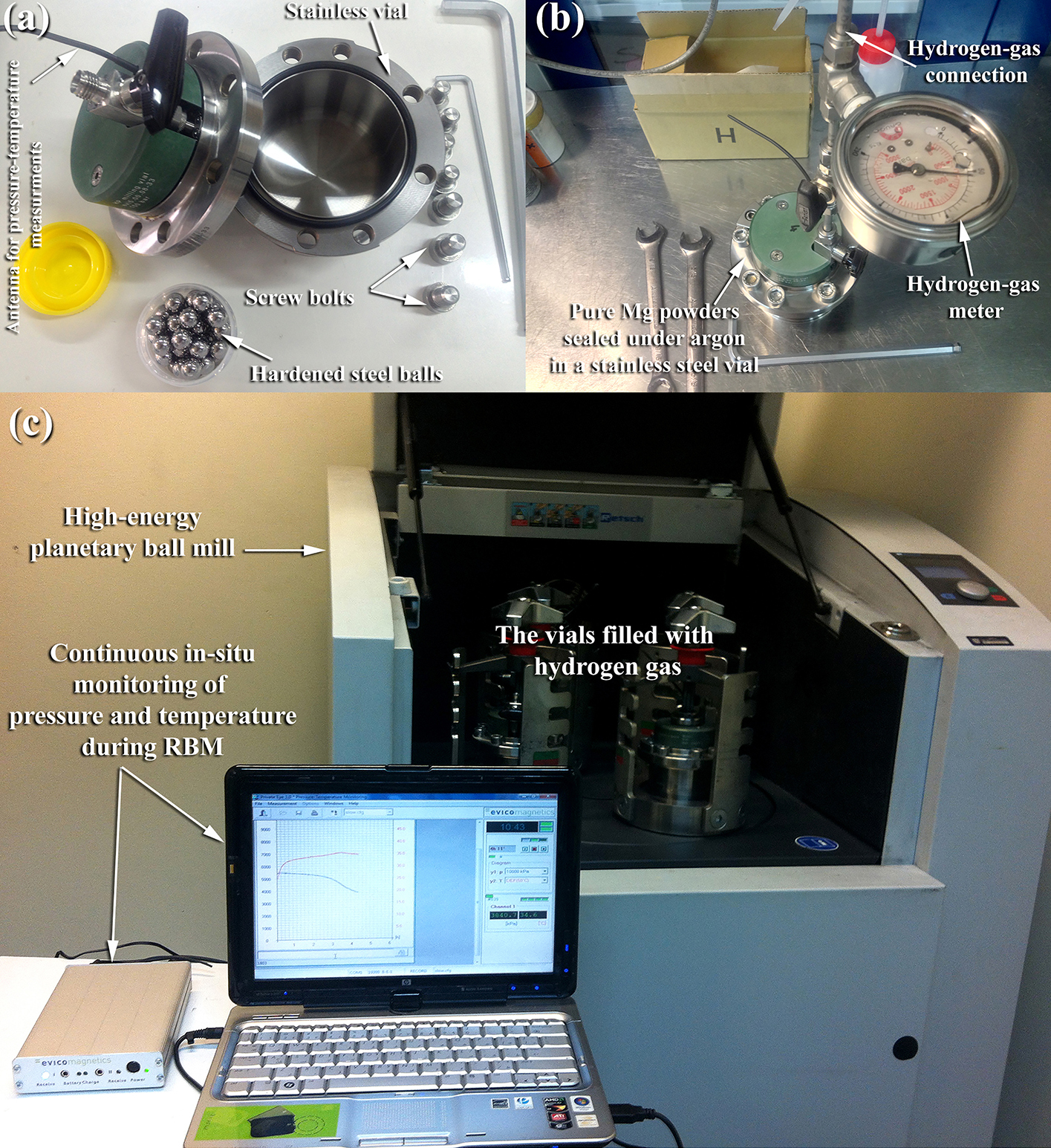


**Supplementary Figure S3**

Photos taken from Nanotechnology Lab in the Energy and Building Research Center, Kuwait Institute for Scientific Research, present (a) the vial and milling media (balls) and (b) the set up performed to charge the ball mill vial with 50 bar of hydrogen gas. The photo in (c) shows the complete set up of GST (supplied by evico magnetic, Germany) system prior to start the reactive ball milling experiment of the present study for preparing of MgH_2_ powders, using Planetary Ball Mill P400, provided by Retsch, Germany).


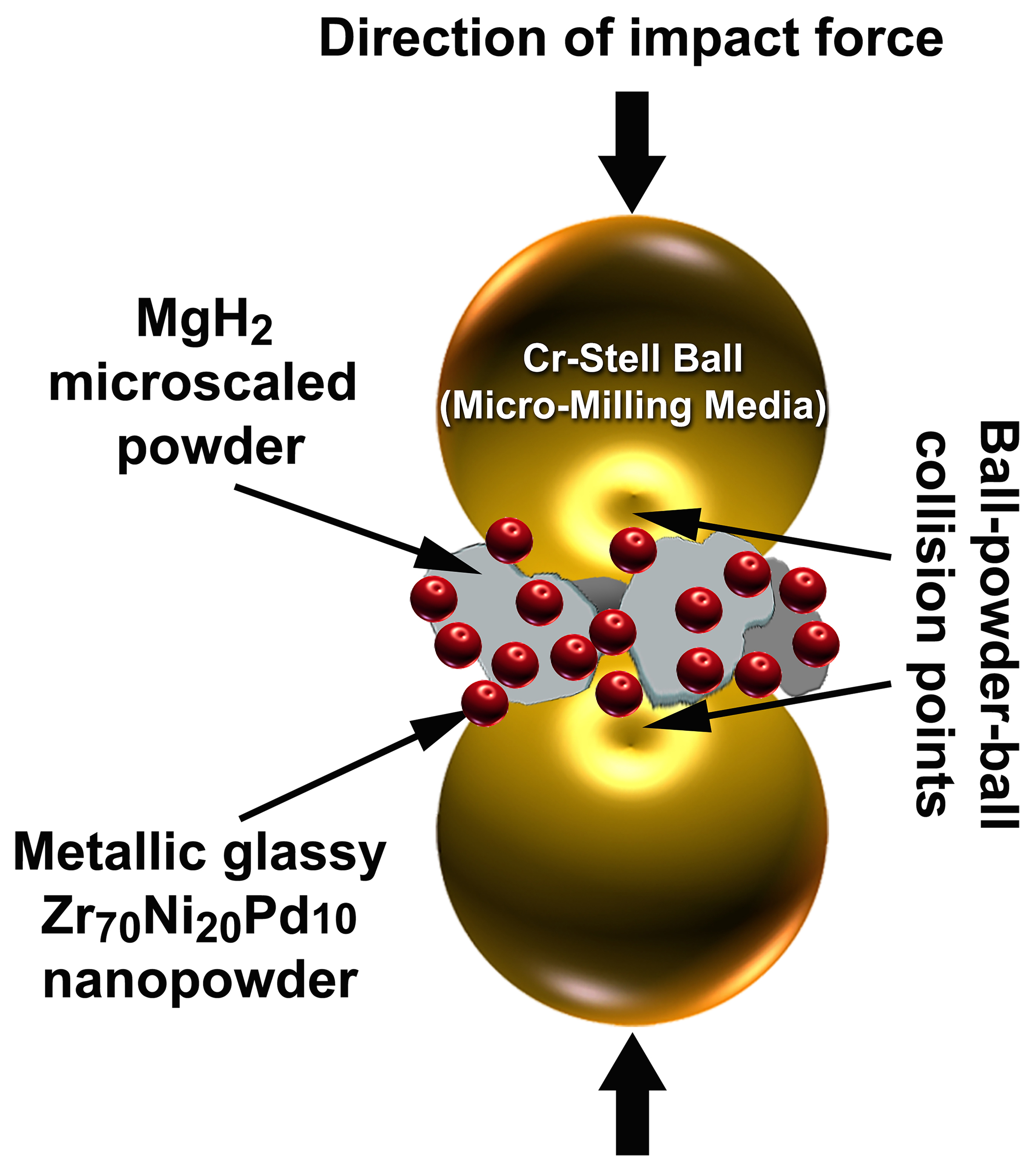


**Supplementary Figure S4**

A schematic illustration presents the a mixture of MgH_2_ and metallic glassy Zr_70_Ni_20_pd_10_ powders that were charged into a stainless steel vial together with stainless steel balls. The powders large MgH_2_ powders were subjected to sever mechanical deformation upon high-speed ball-powder-ball collusions, leading to disintegrate the powders into smaller particles. The red-colored spheres shown in the figure represent the ultrafine metallic glassy powders adhered on the surface of the metal hydride powders.


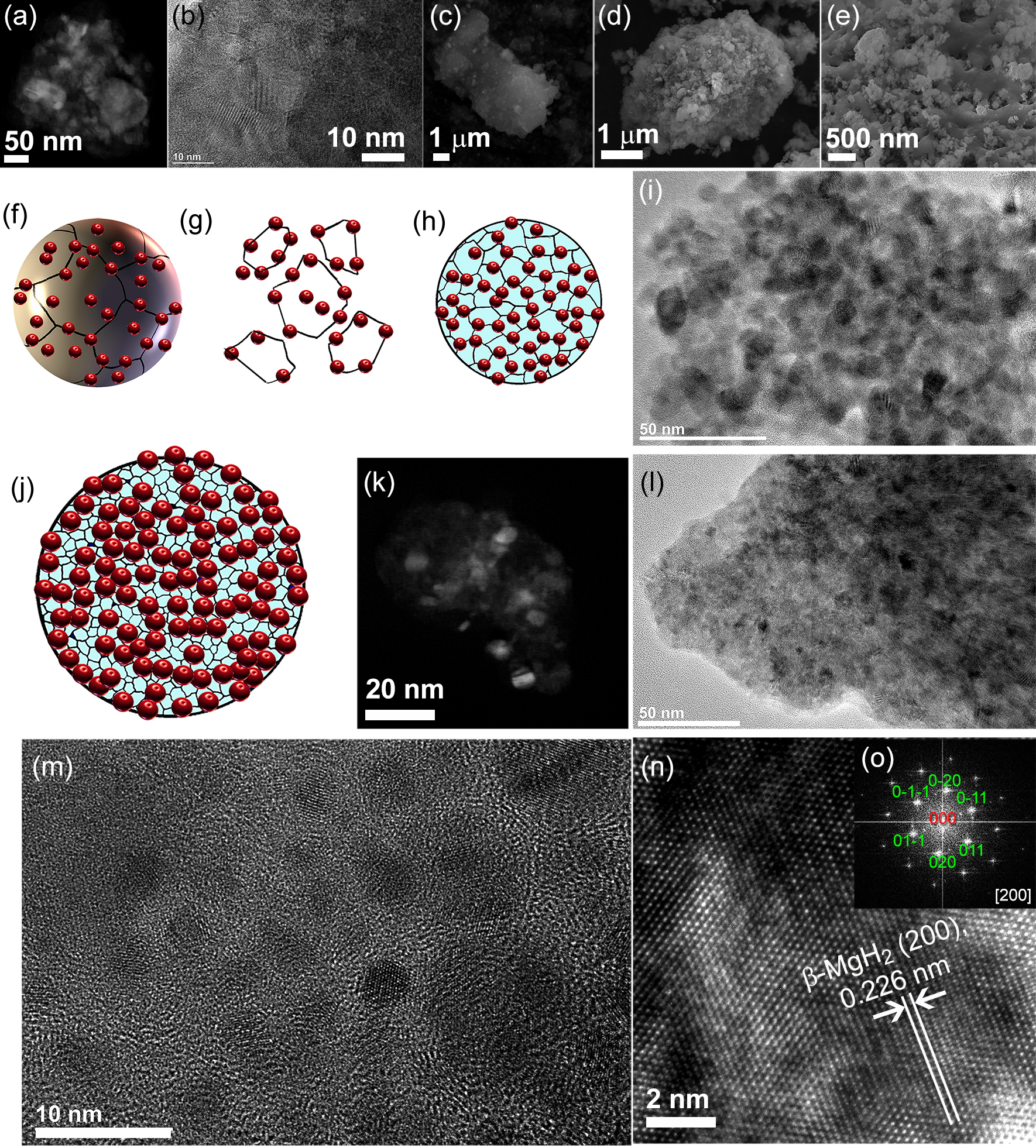


**Supplementary Figure S5**

1. STEM-DFI and (b) HRTEM images of MgH_2_ powders obtained after 6 h of RBM time. The FE-SEM images for MgH_2_/5wt.% and metallic glassy Zr_70_Ni_20_pd_10_ powders obtained after 6, 10 and 20 h of milling are shown in (c), (d) and (e), respectively. The hard particles of the metallic glassy powders immigrated into the core zones of MgH_2_ powders (f) are acting as micro-grain refiner leading to break up the large MgH_2_ grains (g) and to form finer grains (h) of segregated grains (i). Continuous ball time led to further grain refining (j) and the formation of homogeneous nanocomposite powders, as elucidated in (k) and (l). The end-product of MgH_2_/5wt.% metallic glassy Zr_70_Ni_20_pd_10_ powders obtained after 50 h of ball milling (m) possessed homogeneous morphology beyond the nano-scale. The MgH_2_ particles reveal sever defects caused by the applications of mechanical deformation generated by the synergetic actions of milling tools and metallic glassy powders. A typical defected particle oriented to (200) and the corresponding FFT are shown in (n) and (o), respectively.
